# Whole-Genome Sequencing-Based Characterization of *Listeria* Isolates from Produce Packinghouses and Fresh-Cut Facilities Suggests Both Persistence and Reintroduction of Fully Virulent L. monocytogenes

**DOI:** 10.1128/aem.01177-22

**Published:** 2022-10-26

**Authors:** Genevieve Sullivan, Renato H. Orsi, Erika Estrada, Laura Strawn, Martin Wiedmann

**Affiliations:** a Department of Food Science, College of Agriculture and Life Sciences, Cornell University, Ithaca, New York, USA; b Department of Food Science and Technology, Eastern Shore Agricultural Research and Extension Center, Virginia Tech, Painter, Virginia, USA; University of Helsinki

**Keywords:** *Listeria*, *Listeria monocytogenes*, environmental sources, food safety, fresh-cut, packinghouse, persistence, produce, sampling, whole-genome sequencing

## Abstract

The contamination of ready-to-eat produce with Listeria monocytogenes (LM) can often be traced back to environmental sources in processing facilities and packinghouses. To provide an improved understanding of *Listeria* sources and transmission in produce operations, we performed whole-genome sequencing (WGS) of LM (*n* = 169) and other *Listeria* spp. (*n* = 107) obtained from 13 produce packinghouses and three fresh-cut produce facilities. Overall, a low proportion of LM isolates (9/169) had *inlA* premature stop codons, and a large proportion (83/169) had either or both of the LIPI-3 or LIPI-4 operons, which have been associated with hypervirulence. The further analysis of the WGS data by operation showed a reisolation (at least 2 months apart) of highly related isolates (<10 hqSNP differences) in 7/16 operations. Two operations had highly related strains reisolated from samples that were collected at least 1 year apart. The identification of isolates collected during preproduction (i.e., following sanitation but before the start of production) that were highly related to isolates collected during production (i.e., after people or products have entered and begun moving through the operation) provided evidence that some strains were able to survive standard sanitation practices. The identification of closely related isolates (<20 hqSNPs differences) in different operations suggests that cross-contamination between facilities or introductions from common suppliers may also contribute to *Listeria* transmission. Overall, our data suggest that the majority of LM isolates collected from produce operations are fully virulent and that both persistence and reintroduction may lead to the repeat isolation of closely related *Listeria* in produce operations.

**IMPORTANCE** Listeria monocytogenes is of particular concern to the produce industry due to its frequent presence in natural environments as well as its ability to survive in packinghouses and fresh-cut processing facilities over time. The use of whole-genome sequencing, which provides high discriminatory power for the characterization of *Listeria* isolates, along with detailed source data (isolation date and sample location) shows that the presence of *Listeria* in produce operations appears to be due to random and continued reintroduction as well as to the persistence of highly related strains in both packinghouses and fresh-cut facilities. These findings indicate the importance of using high-resolution characterization approaches for root cause analyses of *Listeria* contamination issues. In cases of repeat isolation of closely related *Listeria* in a given facility, both persistence and reintroduction need to be considered as possible root causes.

## INTRODUCTION

Listeria monocytogenes (LM) is a bacterium that can cause serious and sometimes fatal illness. Essentially all listeriosis cases are foodborne and are linked to the consumption of contaminated ready-to-eat (RTE) food products that support LM growth (1, 2). Although LM is also considered soilborne and is frequently found in the natural environment, most listeriosis outbreaks are traced back to contamination from the processing environment in which a product was handled (3). Fresh produce represents a particular challenge, as, unlike many other RTE products (e.g., most dairy, deli meats), production does not include a clear kill step, and contamination of the finished product can consequently originate from sources throughout the supply chain, including irrigation water, fields, field equipment, packinghouses, fresh-cut facilities, and retail. While LM is the foodborne pathogen of concern in the genus *Listeria*, processing facilities, particularly those in North America, use testing for *Listeria* spp. (including LM) to identify conditions that would allow for the introduction, growth, and survival of LM. This approach allows for the identification of “niches” in processing plant environments (i.e., sites that are not adequately addressed during the sanitation process). Thus, there is a need to better understand the transmission of both LM and *Listeria* spp. in the produce supply chain. In this study, we specifically focused on using whole-genome sequencing (WGS) to characterize the transmission of LM and *Listeria* spp. in both packinghouses and fresh-cut facilities.

While molecular subtyping tools (e.g., ribotyping, pulsed-field gel electrophoresis [PFGE]) have been used for more than 2 decades for both foodborne disease surveillance and the characterization of foodborne pathogen isolates from foods and food-associated environments, the last few years have seen a rapid transition to the use of WGS as the preferred tool with which to characterize foodborne pathogen isolates (4). Because of its high discriminatory power, WGS has led to tremendous advances in foodborne outbreak investigations and, therefore, food safety (5). For example, human cases can be more confidently ruled out or ruled in during outbreak investigations using WGS, compared to the former gold-standard method of PFGE, allowing for the prioritization of cases for food history interviews (6). When used for subtyping, WGS data are typically analyzed using either high-quality single nucleotide polymorphism (hqSNP)- based approaches or whole-genome or core genome multilocus sequence typing approaches (abbreviated as wgMLST or cgMLST, respectively) (7–10). In addition, WGS data can be used to rapidly screen for the presence of different genes, including virulence genes (to better predict if a given strain is likely to cause illness) and stress response and sanitizer resistance genes (to help identify appropriate control strategies by which to eliminate persistent LM strains). WGS data can be used to, *in silico*, determine “classical” MLST types, which are based on the sequences for 7 housekeeping gene fragments (11). These sequences can be used to categorize isolates into sequence types (ST), with each ST representing a unique combination of the 7 allelic sequences. STs that differ from one another by a single allelic sequence are further classified into a single clonal complex (CC). In addition to high-resolution, hqSNP-based subtyping, we used the classical MLST approach to characterize the isolates in terms of their ST and CC, as these two classifications are widely used for LM, with several studies showing associations between certain ST or CCs and the presence or absence of genes involved in tolerance to sanitizers, pathogenicity islands, stress survival islets, and virulence (12–14). STs and CCs have also been shown to have distinct associations with food or clinical isolates; for example, studies done in Europe have shown that CC121 is significantly overrepresented among food isolates, whereas CC1 is significantly overrepresented among clinical isolates (15–17). Hence, classification into CCs can provide important insights into the biology and virulence potential of LM isolates.

Molecular subtyping studies of *Listeria* isolates from food processing facilities have been reported for more than 2 decades, with many of these using PFGE (18–20). As public health agencies transition to the use of WGS, there is a need for WGS studies on *Listeria* found in food facilities in order to facilitate the application of this tool to control LM at the source and to gain high-resolution insights into LM transmission. Although a few studies have analyzed isolates from food facilities using WGS (13, 21, 22), many only focused on a single operation (8, 23) or were conducted as follow-ups to an outbreak (24–26). Additionally, there is little information on the WGS-based characterization of *Listeria* from produce operations specifically, including packinghouses and fresh-cut processing facilities, both with regard to species, strain, and virulence diversity and with regard to insights into the transmission of *Listeria* in a given operation (27).

In this study, we analyzed *Listeria* isolates from 16 produce packinghouses and fresh-cut facilities in the United States. The classical 7-gene MLST approach was used for the initial characterization of the isolates, while the United States Food and Drug Administration Center for Food Safety and Applied Nutrition (CFSAN) SNP pipeline was used for high-resolution subtyping. This SNP-based approach is considered at least as discriminatory as the cgMLST approach (7, 8, 13, 28). We categorized the LM isolates by lineage, clonal complex, and sequence type and then screened them for 31 key virulence, stress survival, and resistance genes. Finally, we clustered the *Listeria* isolates, including the non-LM *Listeria* spp. using hqSNP analysis. In doing so, this study details the virulence potential of LM isolates found in produce operations, examines the relatedness of these and other *Listeria* spp. isolates between and within produce operations, and illustrates how WGS can provide evidence for distinct scenarios of *Listeria* transmission in produce operations, including reintroduction and survival over time.

## RESULTS

### Listeria monocytogenes was the most prevalent *Listeria* species among the 16 produce operations.

*Listeria* isolates previously obtained from environmental samples collected in zones 2 to 4 from 16 produce operations, including 13 packinghouses and 3 fresh-cut facilities, were characterized using WGS. This isolate set included 276 isolates designated “representative” (see Materials and Methods for how the representative isolates were defined). The number of isolates sequenced per operation ranged from one (operations VT-I and VT-J) to 59 (operation CU-C) (Table S1). The majority of the isolates were classified as L. monocytogenes (*n* = 169), followed by L. seeligeri (*n* = 47), L. innocua (*n* = 37), L. welshimeri (*n* = 16), L. marthii (*n* = 6), and L. ivanovii (*n* = 1) (Table S1). The 169 LM isolates represented lineage I (*n* = 72), lineage II (*n* = 66), and lineage III (*n* = 31), and they could further be categorized into (i) 41 unique STs, with the largest groups (i.e., >7 isolates) being ST6 (*n* = 12), ST1 (*n* = 11), ST219 (*n* = 11), and ST824 (*n* = 10) as well as (ii) 36 unique CCs, with the largest groups (i.e., >7 isolates) being CC388 (*n* = 15), CC4 (*n* = 13), CC6 (*n* = 12), and CC1 (*n* = 11) (Table S2). A total of 42 LM isolates could not be categorized into an existing ST or CC (Table S2).

In addition to the 276 representative isolates, there were 14 isolates that had been sequenced but were found to be duplicates (i.e., isolates with the same *sigB* allelic type [AT] that were collected from the same site on the same date). These isolates will not be discussed in the remainder of the Results section. However, these isolates, compared against their respective “representative” counterparts, were found to have as many as 4 hqSNP differences, suggesting the potential for highly related but nonidentical isolates to exist in a given sample (Table S3).

### Selected virulence, stress survival, and resistance genes were detected in only a subset of isolates.

**FIG 1:**
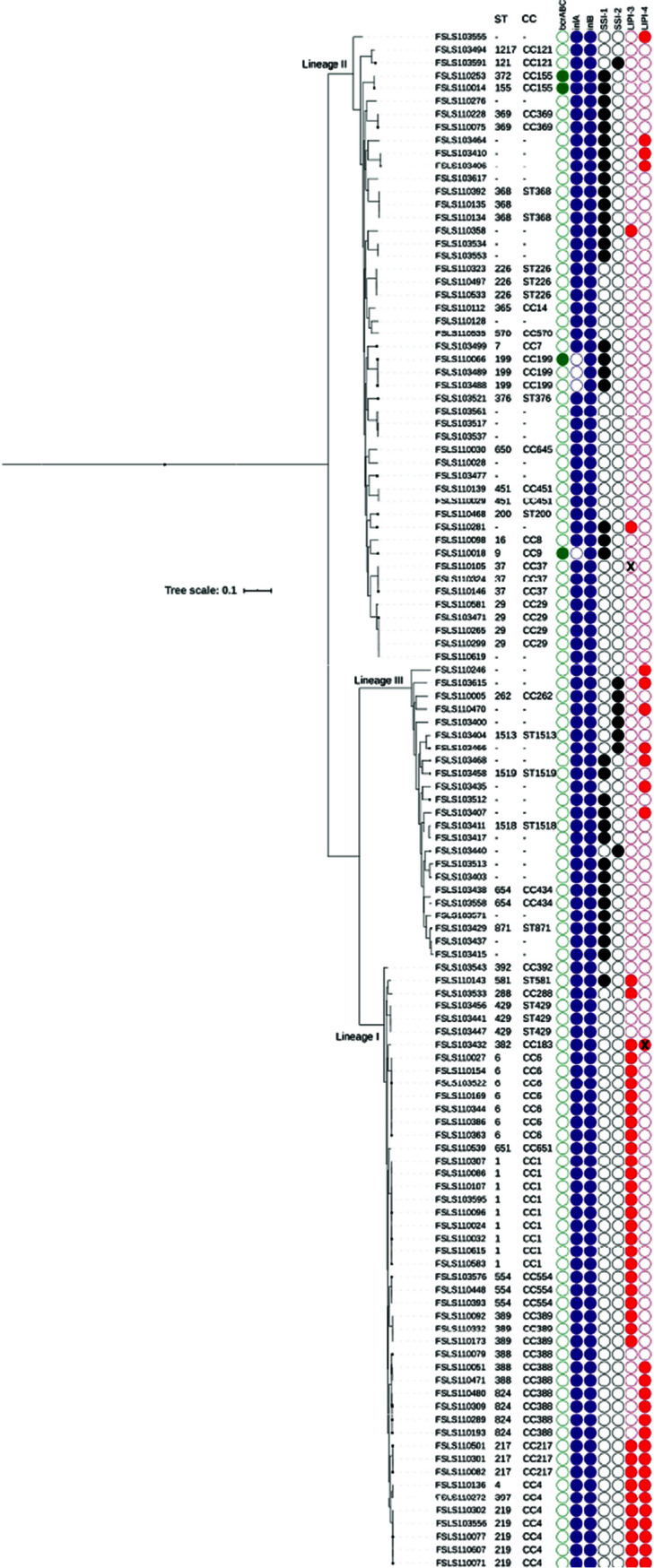
Phylogeny of Listeria monocytogenes isolates based on core single nucleotide polymorphisms (SNPs), annotated with the lineage (shown on the respective branches), clonal complex (CC), sequence type (ST), and presence or absence (indicated by a filled or open circle, respectively) of select genes, including *bcrABC* and *inlB*, as well as the genes in SSI-1, SSI-2, LIPI-3, and LIPI-4. An “X” over an open circle background indicates the partial presence of the locus with less than 50% of the genes present. An “X” over a filled circle background indicates the partial presence of the locus with more than 50% of the genes present. Isolates with premature stop codons in *inlA* are indicated by an open circle. In instances where two or more isolates are identical, only one isolate is shown in the figure.

The genomes of all of the LM isolates were screened for the presence of 31 selected genes with virulence and stress response functions (Fig. 1; Table S2). Additionally, *inlA* was screened for the presence of premature stop codons (PMSC). While all 169 of the LM isolates carried *inlAB*, 9 isolates had *inlA* PMSCs. These 9 isolates grouped into lineage II and one of two clonal complexes: CC199 (7/9) or CC9 (2/9).

For LIPI-3, 58/169 isolates had full matches (>96% similarity over >99% of gene length) to all 8 LIPI-3 genes (Fig. 1; Table S2). In addition, isolate FSL S11-0105 had a full match to 1 gene (>99% coverage with 100% similarity), partial matches (>55% coverage with 100% similarity) to 2 genes, and no matches to the remaining 5 genes from LIPI-3 (see Table S4 for metrics). Therefore, 59/169 isolates were characterized by the full or partial presence of LIPI-3, including 53/72 of the lineage I isolates, 6/66 of the lineage II isolates, and 0/31 of the lineage III isolates (Fig. 1; Table S2). The 59 LM isolates with the full or partial presence of LIPI-3 represented (i) 13 unique STs, with the predominant groups (i.e., >7 isolates) being ST6, ST1, and ST219, and (ii) 11 unique CCs, with the predominant groups (i.e., >7 isolates) being CC4, CC6, and CC1. For LIPI-4, 42/169 isolates had full matches to all 6 LIPI-4 genes (100% similarity over >99% of gene length), with an additional 2 isolates having full or partial matches to all genes (>99% similarity over >49% of gene length). Consequently, 44/169 isolates were characterized by the full or partial presence of LIPI-4, including 32/72 of the lineage I isolates, 5/66 of the lineage II isolates, and 7/31 of the lineage III isolates (Fig. 1; Table S2). The 44 LM isolates with the full or partial presence of LIPI-4 represented (i) 7 unique STs, with the predominant groups (i.e., >7 isolates) being ST219 and ST824, and (ii) 4 unique CCs, with the predominant groups (i.e., >7 isolates) being CC4 and CC388. Overall, 39/169 LM isolates had only LIPI-3, 24/169 isolates had only LIPI-4, and 20/169 isolates had both LIPI-3 and LIPI-4 (considering both complete and partial matches) (Fig. 1; Table S2). Therefore, 86/169 LM isolates had neither LIPI-3 nor LIPI-4.

The stress survival genes for which we screened included those present in the SSI-1 and SSI-2 operons. For SSI-1, 55/169 isolates had full matches (100% similarity over >99% of gene length) to all 5 SSI-1 genes, including 1/72 of the lineage I isolates, 35/66 of the lineage II isolates, and 19/31 of the lineage III isolates (Fig. 1; Table S2). The 55 isolates with the full presence of SSI-1 represented (i) 13 unique STs, with the predominant group being ST199, and (ii) 12 unique CCs, with the predominant group being CC199. However, 24 of these 55 isolates could not be assigned to an existing ST or CC. For SSI-2, 12/169 isolates had full matches to both SSI-2 genes, including 2/66 of the lineage II isolates and 10/31 of the lineage III isolates (Fig. 1; Table S2). These 12 isolates with the full presence of SSI-2 represented 3 unique STs (ST121, ST1513, ST262) and 3 unique CCs (CC121, CC262, and ST1513; ST1513 is the only ST within its respective CC). The majority of isolates with SSI-2 (8/12) could not be assigned to an existing ST or CC. No isolates had both the SSI-1 and SSI-2 operons (Fig. 1; Table S2).

The metal and detergent resistance genes screened included *bcrABC*, *cadAC*, *emrE*, and *qacAH*. Only *bcrABC*, a benzalkonium chloride resistance cassette, was found in the isolates studied here (Fig. 1, Table S2). Of the 169 LM isolates screened, 10 contained *bcrABC*. These 10 isolates all represented lineage II and were classified into one of three clonal complexes: CC155, CC199, and CC9. All 10 of the isolates with *bcrABC* also had the SSI-1 operon.

### hqSNP-based clustering indicates that approximately half of the isolates group into clusters.

The WGS data were also used to perform hqSNP analyses in order to quantify the relatedness of all of the *Listeria* isolates characterized. While previous studies have suggested cutoffs of 20 SNPs (10) or 10 cgMLST alleles (29–31), outbreak-related isolates can differ by >50 hqSNPs (4). Therefore, 3 arbitrary hqSNP cutoffs were used to classify *Listeria* isolates as (i) related (<50 hqSNP differences), (ii) closely related (<20 hqSNP differences), and (iii) highly related (<10 hqSNP differences). Using the hqSNP cutoff of <50 hqSNPs allowed us to group the 276 *Listeria* isolates into 45 clusters (i.e., groups of isolates with <50 hqSNP differences), representing 135 isolates (Table 1) and 141 “singletons”, each of which represents a single isolate that was not related to any other isolate within <50 hqSNPs. The clusters ranged in size from 2 to 10 isolates (Table 1) and represented the species L. monocytogenes (32 clusters), *L. innocua* (5 clusters), *L. seeligeri* (5 clusters), and *L. welshimeri* (3 clusters). Although the majority of the clusters were comprised of isolates from a single operation, 7 clusters contained isolates from 2 operations, and 1 cluster contained isolates from 3 operations (Table 1). A total of 19 LM clusters were comprised solely of isolates obtained from the same operation on the same date but from different sites. An additional 16 clusters included isolates that were obtained from the same facility but on different dates (>60 days apart).

**TABLE 1:** Clusters of isolates with <50 high-quality single nucleotide polymorphism (hqSNP) differences from each other

Cluster number	Operation code(s) of isolates within cluster (number of isolates)	hqSNP range^*a*^	Date range of isolates collected
L. monocytogenes clusters			
1	CU-A (3) CU-C (7) All (10)	CU-A: 0 CU-C: 0 to 37 All: 0 to 39	CU-A: Jan 2018 CU-C: Apr 2018 to Apr 2019 All: Jan 2018 to Apr 2019
2	CU-B (6) CU-C (1) VT-A (3) All (10)	CU-B: 0 to 5 CU-C: NA^*b*^ VT-A: 0 to 1 All: 0 to 30	CU-B: June 2018 CU-C: Dec 2017 VT-A: July 2017 to Aug 2017 All: July 2017 to June 2018
3	CU-A (8)	0 to 5	Oct 2017 to Dec 2017
4	CU-A (4) CU-C (2) All (6)	CU-A: 0 to 1 CU-C: 28 All: 0 to 49	CU-A: Oct 2017 to Jan 2018 CU-C: May 2018 to Apr 2019 All: Oct 2017 to Apr 2019
5	CU-A (3) CU-C (1) All (4)	CU-A: 0 to 15 CU-C: NA All: 1 to 35	CU-A: Oct 2017 to Jan 2018 CU-C: Jan 2018 All: Oct 2017 to Jan 2018
6	VT-C (4)	0 to 4	Sept 2017 to Feb 2018
7	CU-F (4)	3 to 7	Oct 2017 to Oct 2018
8	CU-C (4)	0 to 1	Apr 2018 to May 2018
9	VT-B (3)	1 to 7	Mar 2018
10	CU-D (3)	6 to 11	Aug 2017 to Sept 2018
11	CU-A (3)	0 to 27	Apr 2019
12	VT-D (3)	0	Nov 2017 to Feb 2018
13	CU-A (3)	0	Oct 2017
14	VT-C (3)	2 to 3	Sept 2017 to Nov 2017
15	CU-C (2)	0	Oct 2017
16	CU-C (2)	0	Apr 2019
17	CU-C (2)	0	Apr 2019
18	CU-D (2)	8	Aug 2017 to Sept 2018
19	CU-A (2)	0	Dec 2017
20	VT-A (2)	22	Oct 2017 to Mar 2018
21	VT-A (2)	1	July 2017 to Aug 2017
22	VT-B (2)	1	Mar 2018
23	CU-C (1) CU-E (1)	45	Oct 2017 to Apr 2019
24	CU-A (1) CU-C (1)	42	Oct 2017 to May 2018
25	VT-E (2)	0	Nov 2017
26	VT-F (2)	0	Sept 2017
27	CU-C (2)	27	Dec 2017 to May 2018
28	VT-D (2)	0	Feb 2018
29	VT-C (2)	1	Sept 2017
30	VT-A (2)	6	Aug 2017 to Oct 2017
31	VT-A (2)	3	July 2017 to Aug 2017
32	VT-C (2)	0	Feb 2018
*L. innocua* clusters			
33	VT-B (3)	0	Mar 2018
34	VT-A (3)	3 to 6	Aug 2017 to Oct 2017
35	CU-C (2)	7	Jan 2018 to May 2018
36	VT-F (2)	1	Sept 2017
37	VT-B (2)	6	Mar 2018
*L. seeligeri* clusters			
38	VT-A (2) VT-B (2) All (4)	VT-A: 0 VT-B: 4 All: 0 to 4	VT-A: Aug 2017 VT-B: Aug 2017 All: Aug 2017
39	CU-A (3)	0 to 2	Oct 2017 to Dec 2017
40	CU-A (1) CU-F (1)	19	Oct 2017 to June 2018
41	VT-B (2)	1	Aug 2017
42	CU-C (2)	0	Mar 2018 to May 2018
*L. welshimeri* clusters			
43	CU-C (2)	1	Oct 2017
44	VT-B (2)	17	Aug 2017 to Oct 2017
45	VT-C (2)	0	Nov 2017

a If only one value is provided for the hqSNP range, only one difference exists.

b NA; not applicable. Occurs if there was only one isolate from an operation and therefore there is no within-operation hqSNP difference.

Table S1 provides a list of the metadata associated with each isolate, including a general site description and a cluster assignment for each isolate, if applicable. Of the 276 isolates, 27 isolates (from 10 operations) were collected from equipment frames, with 10/27 of those isolates being part of a cluster, as identified in Table 1. All 10 isolates were <10 hqSNP different from isolates from other sites in a given facility, suggesting a spread to and from equipment frames from other sites in these facilities. This includes 5/10 of these isolates that were <10 hqSNP from an isolate collected from a floor or drain site, demonstrating that *Listeria* found in zone 4 may be an indication that *Listeria* could be present in sites that are in closer proximity to food.

### Analysis of WGS data by operation showed that multiple operations had a re-isolation of highly related isolates at least 60 days apart.

**FIG 2:**
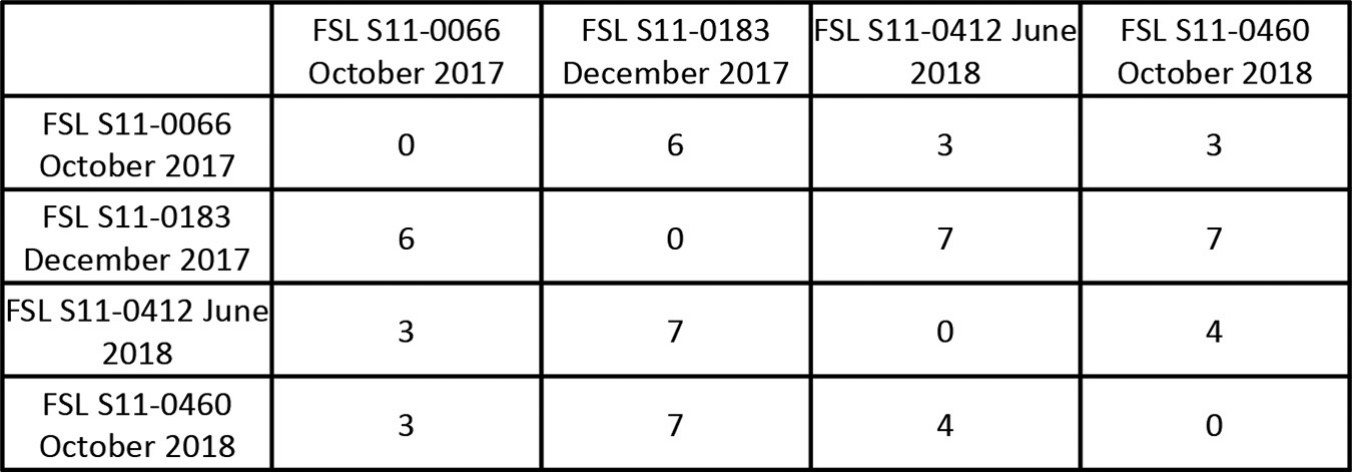
The high-quality single nucleotide polymorphism (hqSNP) distance matrix for Cluster 7, which contains 4 L. monocytogenes isolates from samples collected at operation CU-F over 4 sampling events: October 2017 (FSL S11-0066), December 2017 (FSL S11-0183), June 2018 (FSL S11-0412), and October 2018 (FSL S11-0460).

The analysis of WGS data by operation showed the re-isolation (detection on separate dates and at least 60 days apart) of highly related isolates (<10 hqSNP differences) in 7/16 operations (5 packinghouses and 2 fresh-cut facilities), representing 11/45 clusters. 1, 2, and 4 operations showed evidence for the re-isolation of highly related isolates representing 3, 2, and 1 clusters, respectively. 3 clusters included highly related isolates (<10 hqSNP differences) with dates spanning >1 year. These 3 clusters represented 2 fresh-cut facilities: CU-D (2 clusters) and CU-F (1 cluster). The 2 CU-D clusters (Clusters 10 and 18) (Table 1) were each comprised of isolates found during 2 sampling dates: August 2017 and September 2018. Importantly, no LM was detected during the 5 sample collection events between these 2 collection events. The isolates in Clusters 10 (3 isolates) and 18 (2 isolates) were obtained from trash cans, a trash cart, a pallet jack, and the floor. The locations of these isolation sources indicate that the isolates within Clusters 10 and 18 were dispersed throughout facility CU-D. It is possible that either or both of these strains survived in the operation over the course of the year or that these strains were reintroduced into the operation. In contrast, Cluster 7 (Facility CU-F) (Table 1), included 4 highly related LM isolates (3 to 7 hqSNPs) detected during 4 different sampling events (October 2017, December 2017, June 2018, and October 2018) (Fig. 2). The 4 isolates were from 4 sites in 3 different rooms: an overhead door, a floor/wall juncture, a forklift, and an equipment frame, suggesting a transmission of this strain between zone 2 sites and sites further removed from food contact surfaces. The isolates from the first and last sampling event (October 2017 and October 2018) were found in the same room, which was the product raw receiving area. The product was pre-washed in a neighboring packinghouse before being moved by a trailer to the raw receiving room, suggesting that isolates from this cluster could be reintroduced over time from the transfer trailer or the neighboring operation, neither of which was sampled during this study. Importantly, an investigation of the floor/wall juncture that was positive in December 2017 revealed that the wall had a covering that was used to prevent the original porous wall from getting wet. However, this covering created a sandwich in which moisture could penetrate, creating a potential niche. This was confirmed by follow-up testing done by the operation, which found positive results over repeated samplings of this site. While this wall was removed in January 2018, isolates that fell into Cluster 7 were found again in 2 subsequent sampling events, suggesting that the “Cluster 7 strain” continued to survive in this operation. Additionally, the “Cluster 7 strain” had the *bcrABC* cassette.

**FIG 3:**
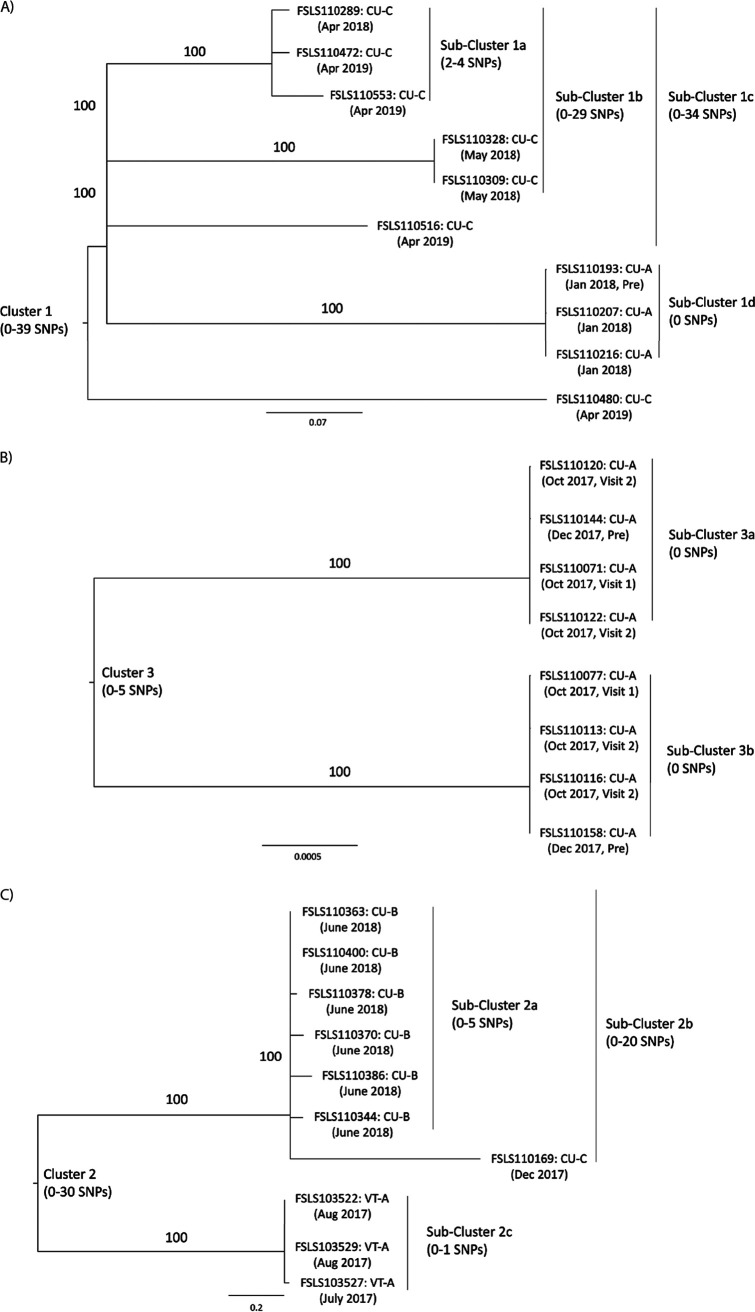
Phylogenetic trees of Clusters 1 (A), 3 (B), and 2 (C). Cluster 1 contains 10 L. monocytogenes isolates from samples collected at 2 different operations, CU-C and CU-A, from sampling events spanning 1 year (April 2018 to April 2019). Cluster 3 contains 8 L. monocytogenes isolates from samples collected at 1 operation (CU-A) over 2 sampling events: October 2017 and December 2017. Cluster 2 contains 10 L. monocytogenes isolates from samples collected at 3 different operations (CU-B, CU-C, and VT-A) during 3 sampling events from July 2017 to June 2018. A designation of “Pre” for the collection date indicates that the isolate was collected during preproduction (i.e., after cleaning but before people and product had begun to move throughout the operation). The hqSNP data for the isolates in this cluster were used to create maximum likelihood phylogenetic trees using RAxML (v 8.2.12) (70) using 1,000 bootstraps and the GTRCAT nucleotide substitution model. Bootstrap values are shown if >70%. Isolate numbers are abbreviated by the removal of spaces and dashes (e.g., FSL S11-0289 is abbreviated as FSLS110289). The hqSNP difference ranges indicated apply to all of the isolates in the cluster or in a given subcluster. The scale on the bottom of the figure indicates genetic distance.

8 clusters (1, 4, 6, 13, 30, 34, 35, and 42) had highly related isolates (<10 hqSNPs) from the same operation obtained between 60 and 365 days apart. 3 of these clusters (1, 35, and 42) included highly related isolates from Packinghouse CU-C. Cluster 35 was comprised of 2 *L. innocua* isolates, while Cluster 42 was comprised of 2 *L. seeligeri* isolates. Cluster 1 isolates from Packinghouse CU-C (Fig. 3A) represented highly related LM isolates found during 2 different packing seasons and included the re-isolation of 2 highly related isolates (2 hqSNPs apart) from the same drain in April 2018 and April 2019 (subcluster 1a; isolates FSL S11-0289 and FSL S11-0472). This drain was located near the end of an approximately 29 m long, uncovered trench drain that spanned 2 rooms (i.e., the drain started in a room and then went underneath a wall to terminate in another room). That second room, which was kept at approximately 26°C (80°F) during production, was where the product was briefly dried. The third isolate within subcluster 1a (FSL S11-0553, 4 hqSNPs apart) was also from this same drain but was further “upstream” in the first room. Cluster 1 also included 3 isolates from Packinghouse CU-A that were collected in January 2018 (all classified into subcluster 1d). These findings are discussed in more detail below. The remaining 5 clusters (Clusters 4, 6, 13, 30, and 34) of highly related isolates (<10 hqSNPs) obtained from the same operation and between 60 and 365 days apart occurred in 4 packinghouses (Table 1).

### WGS comparisons of isolates found preproduction suggest evidence of persistence.

Some of the isolates from Packinghouses CU-A, CU-B, and CU-C were from samples that were collected preproduction (i.e., after cleaning but before the start of production) (Table S1). Preproduction sampling was prompted by repeat positive samples being found at certain sites within the operation. Clusters 1, 3, 4, and 5 all had isolates from CU-A that were collected preproduction. Specifically, Clusters 1 and 5 included isolates from preproduction samples that were highly related (0 to 1 hqSNPs) to isolates from samples collected during the following production shift. Cluster 4 included isolates collected during production in October 2017 and January 2018. These isolates were highly related (1 hqSNP) to an isolate collected preproduction in December 2017. Most notably, Cluster 3 (Fig. 3B) had 2 highly related subclusters (3a and 3b; 5 hqSNP differences between clusters) (Fig. 3B) which each represented isolates obtained from a specific area of the facility. Each of these 2 subclusters included 3 isolates collected during production in October 2017 and 1 isolate collected preproduction in December 2017 (Fig. 3B). For each subcluster, all 4 isolates showed differences of 0 hqSNPs. These data suggest that these 2 strains (subclusters 3a and 3b) are surviving standard sanitation protocols and are persisting in the operation over time. This is supported by the fact that these isolates not only were found on separate sampling events in different months but also were found preproduction (which occurs after sanitation but before people and product begin moving through the operation). These isolates did not have the *bcrABC* cassette.

### Highly related isolates were found in separate, independently owned operations.

Among all 45 hqSNP clusters, 8 represented isolates from multiple operations (Clusters 1, 2, 4, 5, 23, 24, 38, and 40), with 2 clusters (Clusters 2 and 40) having isolates from separate operations with <20 hqSNP differences and 1 additional cluster (Cluster 38) having isolates from separate operations with <10 hqSNP differences. Among the 8 clusters with isolates from multiple operations, 6 represented LM clusters. Only 1 LM cluster (Cluster 2) with isolates from multiple operations had isolates from separate operations with <20 hqSNP differences. More specifically, subcluster 2b included 6 isolates from Packinghouse CU-B (all obtained in June 2018) and 1 isolate from Packinghouse CU-C (obtained in December 2017), and these differed from the CU-B isolates by as few as 17 hqSNPs (Fig. 3C). This represented the least hqSNP difference between LM isolates from 2 operations for this study. These 2 packinghouses were located in the same region of the United States and packed the same commodity. These findings suggest a potential common upstream source, as opposed to these strains persisting in each operation over time. A third operation (VT-A) also had 3 isolates that were part of this cluster (subcluster 2c), These isolates were collected in July and August 2017 and differed from all of the other isolates in cluster 2 by >25 hqSNPs. The remaining 5 LM clusters with isolates from multiple operations ranged from 30 to 49 hqSNP differences between isolates from separate operations, potentially representing *Listeria* that are more broadly distributed in the environment.

**FIG 4:**
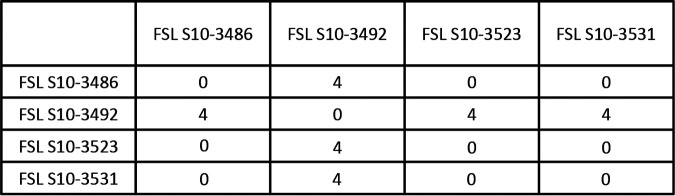
The hqSNP distance matrix for Cluster 38, which contains 4 Listeria seeligeri isolates from samples collected on the same date (August 2017) from operations VT-A (FSL S10-3523 and FSL S10-3531) and VT-B (FSL S10-3486 and FSL S10-3492).

The 2 non-LM clusters with isolates from multiple operations both represented *L. seeligeri*, and both contained isolates from separate operations with <20 hqSNP differences (Table 1). Cluster 40 had 2 isolates (1 from Packinghouse CU-A and 1 from Fresh-cut Facility CU-F) that were 19 hqSNP apart. These two operations were located in the same region of the United States. Cluster 38 (Table 1) included 4 isolates with 3 identical *L. seeligeri* isolates (0 hqSNP difference) isolated from samples collected in 2 packinghouses (VT-A and VT-B) on the same day. Sample collection was performed by different personnel associated with each operation (Fig. 4), and the 4 isolates within this cluster were collected from 4 different sites. Although the authors acknowledge the possibility of cross-contamination within the lab, the positive-control used throughout the study was a strain of LM, whereas Cluster 38 was comprised of *L. seeligeri* isolates. In addition, all of the negative-controls were negative throughout the study. Moreover, the samples from the two packinghouses in this instance were processed by two different people (i.e., one person processed all of the samples collected from the first packinghouse, and a different person processed the samples from the second packinghouse). Further investigation revealed that in addition to being geographically close (<20 miles), it was not uncommon for these two packinghouses to employ the same workers, who resided at the same housing site for temporary, nonimmigrant workers, for different shifts (e.g., one employee may help with packing in the morning at one operation and with sanitation in the evening at the second operation), suggesting that shared employees could have played a role in cross-contamination between the operations. This possibility is supported by an *L. seeligeri* isolate being detected in a sample collected from the employee breakroom of one of the packinghouses (Packinghouse VT-B, isolate FSL S10-3508), although the isolate did not cluster with any of the other detected isolates.

## DISCUSSION

### L. monocytogenes in produce operations are likely to have the ability to cause human disease.

Overall, our data suggest that a large proportion of the LM isolates obtained from the United States produce operations included in this study have the ability to cause human disease, as supported by (i) the classification of a considerable number of isolates into hypervirulent and outbreak-associated CCs, (ii) the frequent presence of LIPI-3 and LIPI-4, and (iii) the infrequent presence of strains with virulence attenuated *inlA* PMSCs. More specifically, the most frequent CC (CC388), which represented 15 of the 169 LM isolates in this study, was associated with a pork-related outbreak that sickened more than 200 people in Spain in 2019 (32, 33). The 3 next most frequent CCs from this study represented CC4 (*n* = 13), CC6 (*n* = 12), and CC1 (*n* = 11), which have also been associated with human illness (15, 34–38) and have all been suggested to represent hypervirulent LM (15). In addition to other outbreaks (38, 39), CC4 has been associated with a 2013 outbreak in Switzerland that was linked to salad (39). We also identified two CC7 isolates, and this CC was associated with the 2011 United States cantaloupe outbreak (39), indicating that CCs that have previously been associated with produce related outbreaks continue to be found in produce processing facilities.

Only 9/169 (5%) of the LM isolates in this study were found to have *inlA* PMSCs, which have been shown to result in virulence attenuation of LM isolates due to their low invasion efficiency into intestinal epithelial cells (40, 41). These isolates were categorized into two CCs that have historically been isolated from food sources rather than from clinical sources: CC199 and CC9 (15, 42). More specifically, a previous study of 6,633 isolates from France, including 2,584 clinical isolates, classified CC9 as a food-associated clone that rarely causes human disease (15). A different study of 300 LM isolates (117 clinical) from 5 continents included 6 isolates from CC199, which were all from food or environmental sources (42). Our finding that only 5% of the LM isolates in our study had *inlA* PMSCs is in contrast to previous studies that suggest that *inlA* PMSCs are common among isolates from RTE foods, processing plants, and retail environments (40, 41, 43–45). For example, Van Stelten et al. found that 45% of 502 isolates from RTE foods (i.e., bagged salads, fresh soft cheeses, soft ripened cheeses, smoked seafood, seafood, and deli salads and meats) carried an *inlA* PMSC, compared to 5% of human clinical isolates (*n* = 507) (43).

Several of the isolates included in this study had full or partial matches to the pathogenicity islands LIPI-3 (59/169) or LIPI-4 (44/169), including 20/169 isolates with matches to both. Both LIPI-3 and LIPI-4 have been shown to be associated with hypervirulence (15, 46). Overall, LIPI-3 was found in 35% of the LM isolates characterized in our study. By comparison, Chen et al. (12), found that 25% of the 102 LM isolates (recovered from 27,389 United States refrigerated RTE food samples) carried LIPI-3, while Kim et al. (14) found that 37% of 121 LM isolates (recovered from milk, milk filters, and milking equipment on bovine dairy farms) had LIPI-3. However, Hurley et al. (13) reported that only 10% of 100 LM lineage I and II isolates from food processing environments carried LIPI-3. More specifically, LIPI-3 was found among 74%, 9%, and 0% of the lineage I, II, and III LM isolates, respectively, that were characterized here. While LIPI-3 is generally thought to be restricted to lineage I (29), previous studies (13, 14) have found at least one lineage II isolate that had partial matches to LIPI-3. The frequencies of LIPI-3 per lineage found in our study (74%, 9%, and 0% of lineages I, II, and III, respectively) were higher than or consistent with those reported in previous studies. Kim et al. (14) found that 73%, 2%, and 0% of lineage I, II, and III isolates had LIPI-3, respectively. Hurley et al. (13) found that 39% and 1% of lineage I and II isolates had LIPI-3, respectively. Chen et al. (12) found that 51% of the lineage I isolates, but none of the lineage II or III isolates, had LIPI-3.

Overall, LIPI-4 was found in 26% of the LM isolates characterized in our study. This is higher than the percentages reported by Kim et al. (14), Chen et al. (12), and Hurley et al. (13), who found that 17%, 15%, and 1% of isolates had LIPI-4, respectively. More specifically, our study identified LIPI-4 in 44%, 8%, and 23% of lineage I, II, and III isolates, respectively. Kim et al. (14) found LIPI-4 among 32%, 0%, and 33% (1 isolate) of lineage I, II and III isolates, respectively. Chen et al. (12) found LIPI-4 among 30.6% of lineage I isolates and none of the lineage II or III isolates in their study. Hurley et al. (13) found LIPI-4 in 4% of lineage I isolates and in no lineage II isolates. The fact that we identified LIPI-4 among lineage II isolates is surprising and may require further follow-up studies (e.g., long range sequencing) to confirm the presence of LIPI-4, determine the genomic location of LIPI-4, and probe for possible lateral transfer events that may have introduced LIPI-4 into lineage II strains.

There was a surprisingly high proportion of isolates in this study (31/169) that were categorized as lineage III, which has been historically found to be underrepresented among isolates from human clinical cases (47). However, lineage III has also been considered to be underrepresented among isolates from foods and instead appears to be more commonly associated with food-production animal sources, particularly ruminants (47, 48), with a recent report also indicating a high prevalence of lineage III isolates among isolates collected from natural environments across the United States (49). This could suggest that a considerable proportion of produce-associated isolates could come from an animal source or a yet to be identified source of lineage III isolates that is common to farm animals and produce operations.

### While a number of L. monocytogenes isolates from produce operations are likely to have stress response islands, few contain genes that convey reduced metal, detergent, or quaternary ammonium sensitivity.

In total, there were slightly fewer isolates that had either SSI-1 (55/169) or SSI-2 (12/169), compared to the proportions of isolates with LIPI-3 or LIPI-4. Various studies have shown a diverse range of occurrence of SSI-1 among LM isolates characterized, ranging from 33% to 70% (12–14, 50–53). When comparing within lineages, our data showed that 1%, 53%, and 61% of lineage I, II, and III isolates had SSI-1, respectively. In comparison, Chen et al. (12) found SSI-1 in 35%, 80%, and 100% of lineage I, II, and III isolates, respectively, while Kim et al. (14) found SSI-1 in 47%, 43%, and 33% of lineage I, II, and III isolates, respectively. The 33% SSI-1 prevalence among the isolates characterized here is lower than what was reported by Chen et al. (12), Hurley et al. (13), and Kim et al. (14), who found that 57%, 51%, and 45% of isolates had SSI-1, respectively. For SSI-2, our data showed that 0%, 3%, and 32% of lineage I, II, and III isolates had SSI-2, respectively. Fewer studies appear to screen isolates for the SSI-2 operon, with most studies showing few isolates (0 to 5%) having the operon (12, 13, 52, 53) and Hurley et al. (13) showing 12% of isolates (16% of lineage II isolates) having SSI-2. Overall, compared to previous studies, our study found a lower frequency of LM isolates with SSI-1 (12–14, 50–53) but a higher frequency of LM isolates with SSI-2 (12, 13, 52, 53), possibly due to the fact that the isolates characterized here included a larger number of lineage III isolates.

Our study found that only a few isolates contained any of the selected genes we screened for from the “metal and detergent resistance” category (see Materials and Methods). The only genes detected in our isolates were those representing the *bcrABC* resistance cassette, which has been shown to confer reduced sensitivity to a quaternary ammonium (“quat”) compound called benzalkonium chloride, which is commonly used for sanitation in food production environments. *bcrABC* was found in 10/169 (6%) of the LM isolates studied here, including all 4 isolates from Cluster 7, which appear to have persisted in facility CU-F. The 10 isolates with *bcrABC* found here were all from lineage II (10/66, 15%), and the proportion of isolates with *bcrABC* is lower than what was found by Chen et al. (12), who found this cassette in 10/49 (20%) and 35/51 (69%) of lineage I and lineage II isolates, respectively. For the produce-specific isolates within that study (12), 2/14 (14%) and 9/14 (64%) of the lineage I and II isolates had *bcrABC*, respectively. A study investigating 100 LM isolates from three meat and vegetable processing facilities found that 19% of the isolates had the *bcrABC* cassette (13). A different study that characterized 15 produce-associated LM isolates in the United Kingdom found that 2/15 (13%) isolates had *bcrABC*. Our data suggest that *bcrABC* presence may be less common in isolates found in the produce-associated operations studied here, compared to previous studies. This could at least be partially due to the fact that a considerable proportion of the LM isolates characterized here (i.e., 91%) were obtained from packinghouses, which may be less likely to use quaternary ammonium compounds. Additionally, it is important to note that *cadAC*, *emrE*, and *qacAH* were not detected in any of the isolates studied here. Overall, our findings are consistent with those reported in previous studies (54, 55) that have not identified a strong association between the presence of specific “persistence” genes. While the findings to date could suggest that the establishment of persistence may include a strong element of chance (meaning persistence is likely to occur when an appropriate strain is introduced into a facility location that represents a potential niche where *Listeria* would not be removed by sanitation), further studies that use even larger isolate sets than those described here and tools such as genome-wide association to identify new genetic markers that are putatively associated with persistence would be valuable. In addition to the large sample sizes needed for these types of studies, a continued challenge with these types of studies will be classifying isolates as truly “sporadic”. Isolates may be misclassified as sporadic if they persist in locations that are difficult to sample or are not sampled for other reasons.

### Both sporadic and persistent *Listeria* spp. and LM contribute to the environmental contamination of produce facilities.

In addition to 141 isolates that did not fall into any hqSNP cluster, we also found that 19/45 clusters in this study (representing 42 isolates) were comprised of isolates from a single operation obtained on a single date but from different sites. Hence, the majority of *Listeria* or LM positive sites appear to be due to sporadic contamination, with some representing short-term *Listeria* spread within an operation, with contamination apparently controlled via standard cleaning and sanitation practices that were in place. However, our data showed the re-isolation (detection on separate dates that are at least 60 days apart) of highly related isolates (<10 hqSNP differences) in 7/16 operations (5 packinghouses and 2 fresh-cut facilities), suggesting that persistent contamination (or reintroduction, as discussed further below) is still frequent among United States produce operations (Table S5). Additionally, our data indicate that persistent *Listeria* contamination can occur in both packinghouses and fresh-cut facilities. This is consistent with previous studies that found that a significant proportion of food-associated operations show evidence for LM or *Listeria* persistence. For example, in a study of 9 small cheese processing facilities, 7 facilities showed evidence for *Listeria* spp. persistence (20). Similarly, in a study of 30 deli operations, 12 showed evidence for persistence (18). More specifically, we identified 6 LM clusters that had isolation dates spanning >1 year, providing evidence for long-term persistence. This includes 3 clusters (representing 2 fresh-cut facilities) that had isolates from a single operation that were highly related (<10 hqSNPs) and detected >1 year apart. Importantly, these 2 fresh-cut facilities were operated continuously, whereas the packinghouses in this study, from which data were collected over >1 year, were operated seasonally. Therefore, the packinghouses all had an “off-season” in which equipment was down and able to be disassembled, cleaned, and dried for an extended period. This could potentially explain why we did not detect highly related isolates among those collected >1 year apart in any of the participating packinghouse operations, which is consistent with the findings of a previous study that found low LM prevalence and no evidence for persistence in 2 seasonally operated crawfish processing facilities (56). Overall, our findings are also consistent with prior observations that LM strains may survive in operations for extended periods of time, as supported by a study that showed a processing plant that had a single strain persisting over at least 12 years (57). Interestingly, the fact that a single produce processing facility included 3 distinct LM strains that showed evidence for persistence (Packinghouse CU-C had 3 clusters of highly related isolates found between 60 and 365 [exclusive] days apart) suggests that some facilities may be more prone to allowing for the establishment of persistence. Based on observations of this operation, persistence may occur due to equipment with poor sanitary design or infrequent sanitation procedures, and these observations are in concordance with a previous review, which found that one of the two most common risk factors for persistence mentioned in the literature was equipment cleanability (58). However, we did not formally assess here which specific factors may have the greatest impact on the likelihood of persistence occurring in a given facility. While future studies on risk factors for persistence will be valuable, they will be challenging, as a large number of facilities would need to be enrolled.

### Evidence of cross-contamination or common sources that contribute similar *Listeria* spp. and LM in multiple facilities.

While, as discussed above, the repeat isolation of closely related *Listeria* often is interpreted as providing evidence for persistence, these types of findings could also be due to reintroduction from outside or upstream sources that are persistently contaminated with a given *Listeria* strain. Interestingly, we found that 7 of the 45 hqSNP clusters in this study were comprised of isolates from 2 operations. An additional cluster (Cluster 2) included isolates from 3 operations, with all isolates within this cluster differing by <30 hqSNPs. Our findings that 8 clusters contained isolates from at least 2 operations demonstrate the potential for closely related isolates to be collected at different operations and provides evidence of the introduction of specific *Listeria* strains into multiple facilities from a common source. More specifically, 1 cluster showed LM isolates that were as few as 17 hqSNPs apart and were collected from 2 packinghouses. This could be due to a common source of raw materials obtained from facilities or fields that harbor this strain or could represent a widespread presence of isolates representing this given hqSNP cluster in the environment. Interestingly, we also identified *L. seeligeri* isolates that had 0 hqSNP differences in 2 different packinghouses that shared employees, despite being separately owned and operated. This provided a potential mechanism for cross-contamination between facilities. In concordance with our findings, other studies have also identified closely related *Listeria* spp. isolates from different facilities, although it is important to note that different SNP-based data analysis approaches may not be directly comparable (59). For example, 1 study showed that LM isolates with <10 SNP differences were isolated from multiple delis in separate states in the United States (22), suggesting the introduction of closely related LM into multiple facilities, likely from an upstream source, such as a common supplier. Another study, which investigated isolates from a cold-smoked salmon facility, showed that an isolate from a different cold-smoked salmon facility was within 11 to 23 SNPs of the other isolates within the cluster (8). Other studies have shown instances in which closely related isolates have been associated with separate operations, such as an LM strain that was tied to two ice cream production facilities, one of which purchased ingredients from the other (25). Our findings further support the importance of using WGS data in combination with metadata to help differentiate between re-contamination and persistence. For example, the repeat isolation of closely related *Listeria* isolates after sanitation and preoperation in locations with limited traffic during off hours (e.g., production rooms) supports persistence, while the repeat isolation of closely related *Listeria* isolates only during operations and from sites close to potential introduction routes (e.g., a receiving dock), more likely indicates re-contamination. In addition, advanced WGS data analysis, including the construction of tip-dated phylogenies (see Harrand et al. for an example [60]) can provide information on the most recent common ancestor (MRCA) of closely related isolates. In this case, an MRCA that predates the construction date of a facility could also suggest a reintroduction rather than persistence (particularly if supported by metadata). Importantly, if closely related isolates are found in different facilities, this may also suggest contamination from higher-up in the food supply chain (e.g., agricultural water, fields, field equipment) or from common employees, for example. Re-introduction may be more likely in supply chains where no kill steps (e.g., heat treatment) are applied (e.g., fresh produce), which would facilitate survival throughout the supply chain. Our study specifically shows that the availability of larger WGS data sets that are comprised of isolates from multiple facilities, along with detailed root cause and epidemiological approaches, will help to differentiate persistence from reintroduction or cross-contamination. This will help facilities more rapidly address the true root causes of contamination events.

## MATERIALS AND METHODS

### Isolate selection.

*Listeria* isolates were obtained from two previous studies (61, 62) that investigated *Listeria* prevalence in 13 produce packinghouses (VT-A, VT-B, VT-C, VT-D, VT-E, VT-F, VT-G, VT-H, VT-I, VT-J, CU-A, CU-B, CU-C) and 3 fresh-cut facilities (CU-D, CU-E, CU-F) (61, 62). These two studies collected a total of 4,152 environmental samples, of which 217 were positive for *Listeria*. From these 217 *Listeria*-positive samples, a total of 679 *Listeria* isolates were collected. Additionally, a third study collected 156 environmental samples from 3 of the operations that participated in the Sullivan et al. (62) study (i.e., operations CU-A, CU-C, CU-E) approximately 1 year after the original study, resulting in 21 *Listeria*-positive samples and 155 *Listeria* isolates. Only LM isolates from the third study were included in the study reported here. To identify unique representative isolates from each sample, *sigB* allelic typing was performed on all of the isolates as previously described (63). One isolate of each *sigB* allelic type that was present in a given sample was considered “representative”. Therefore, a single positive sample could have more than one representative isolate if these isolates showed distinct *sigB* allelic types. Additionally, different representative isolates could have the same *sigB* allelic type, as long as they originated from different samples. These “representative” isolates (*n* = 276) were characterized via whole-genome sequencing, as detailed below. In certain instances, such as when preliminary sequencing data were unavailable at the time of strain selection, the whole-genome sequencing of an isolate that was ultimately designated representative was not performed. Operation code designations for each isolate are consistent with those of the original studies. However, to differentiate between studies, the operations studied by Sullivan et al. were given a “CU” prefix (i.e., Cornell University), and the operations studied by Estrada et al. were given a “VT” prefix (i.e., Virginia Tech) (61, 62).

### Whole-genome sequencing.

The total DNA was extracted from representative isolates for whole-genome sequencing as previously described (22). Sequencing was performed using a HiSeq 2500 (Illumina, Inc., San Diego, CA, United States) with a maximum read length of 2 × 150 bp. Due to the sensitivity of the research reported here and to protect the data privacy of the participating operations, the sequences were not uploaded to NCBI. The assemblies, however, are publicly available on the Cornell University eCommons repository: https://doi.org/10.7298/74sp-fg52 (64). Genomes were assembled as previously described (22). Briefly, Trimmomatic (v 0.39) was used to trim and filter the raw reads (65), which were then evaluated based on quality using FastQC (v 0.11) (66). The trimmed reads were assembled using SPAdes (v 3.13) (67) with k-mer sizes of 21, 33, 55, and 77 bp. All of the isolates, including the non-LM *Listeria* species, were sorted into groups based on *sigB* allelic type before performing a reference-free SNP analysis using kSNP3 (v 3.1) (68) to determine the clusters for the hqSNP analysis. Using the results of the kSNP3 analysis, isolates that were <100 SNPs apart were then analyzed using the CFSAN SNP pipeline (v1.0.1) (69) to determine high-quality single nucleotide polymorphisms (hqSNPs). The reference assembly for the United States Food and Drug Administration CFSAN SNP pipeline analysis was selected to represent the isolate with the maximum Q value within each cluster. The Q value was obtained via the following equation.

Q = [Total Genome Length − (Number of Contigs)*1000]

If, after the hqSNP analysis, clusters had isolates that were >50 hqSNPs away from all of the other isolates, the isolate was removed, and the cluster was reanalyzed. Therefore, if a cluster had 2 or more subclusters that were >50 hqSNPs apart, the cluster was split into multiple subclusters for analysis. Maximum likelihood phylogenetic trees based on the hqSNP data for select clusters were created using RAxML (v 8.2.12) (70) with 1,000 bootstraps and the GTRCAT nucleotide substitution model.

### Gene presence or absence in LM.

To assess whether virulence and stress-response genes were present in the genomes sequenced here, allele sequences were downloaded from the Pasteur Institute′s BIGs-LM database from the following schemes: (i) Virulence (all 8 genes in LIPI-3 and all 6 genes in LIPI-4), (ii) Metal & Detergent Resistance (*bcrABC*, *cadAC*, *emrE*, and *qacAH*), and (iii) Stress Islands (all 5 and 2 genes in SSI-1 and SSI-2, respectively) (29, 71–73). Additionally, the classical 7-gene MLST scheme (*abcZ*, *bglA*, *cat*, *dapE*, *dat*, *idh*, *ihkA*) was downloaded from the Pasteur Institute’s BIGs-LM database for the *in silico* MLST of the isolates using the WGS data. A database was created of the seven MLST genes, which were used to assign each LM isolate to a sequence type (ST) and a clonal complex (CC). The *inlA* and *inlB* alleles were also downloaded from the Pasteur Institute’s BIGs-LM database, and a BLAST database was created to determine whether each gene was present or absent. MEGA X (v 10.1.7) (74) was used to identify isolates with PMSCs in *inlA*. The presence or absence of genes and the presence of *inlA* PMSCs were annotated, using iTOL (v1.0) (75), in a phylogenetic tree that was generated from the kSNP3 analysis of all of the LM isolates.

## References

[1] U.S. Food and Drug Administration, Office of Foods and Veterinary Medicine Center for Food Safety and Applied Nutrition. 2017. Draft guidance for industry: control of *Listeria monocytogenes* in ready-to-eat foods. Available at: https://www.fda.gov/regulatory-information/search-fda-guidance-documents/draft-guidance-industry-control-listeria-monocytogenes-ready-eat-foods.

[2] Centers for Disease Control and Prevention (CDC). 2018. The Listeria initiative | Listeria | CDC. Available at: https://www.cdc.gov/listeria/surveillance/listeria-initiative.html. Retrieved 28 May 2020.

[3] Centers for Disease Control and Prevention (CDC). 2020. Listeria Outbreaks | Listeria | CDC. Available at: https://www.cdc.gov/listeria/outbreaks/index.html. Retrieved 28 May 2020.

[4] Jackson BR, Tarr C, Strain E, Jackson KA, Conrad A, Carleton H, Katz LS, Stroika S, Gould LH, Mody RK, Silk BJ, Beal J, Chen Y, Timme R, Doyle M, Fields A, Wise M, Tillman G, Defibaugh-Chavez S, Kucerova Z, Sabol A, Roache K, Trees E, Simmons M, Wasilenko J, Kubota K, Pouseele H, Klimke W, Besser J, Brown E, Allard M, Gerner-Smidt P. 2016. Implementation of nationwide real-time whole-genome sequencing to enhance listeriosis outbreak detection and investigation. Clin Infect Dis 63:380–386. 10.1093/cid/ciw242. 27090985 PMC4946012

[5] Pietzka A, Allerberger F, Murer A, Lennkh A, Stöger A, Cabal Rosel A, Huhulescu S, Maritschnik S, Springer B, Lepuschitz S, Ruppitsch W, Schmid D. 2019. Whole genome sequencing based surveillance of *L. monocytogenes* for early detection and investigations of listeriosis outbreaks. Front Public Health 7:139. 10.3389/fpubh.2019.00139. 31214559 PMC6557975

[6] Angelo KM, Conrad AR, Saupe A, Dragoo H, West N, Sorenson A, Barnes A, Doyle M, Beal J, Jackson KA, Stroika S, Tarr C, Kucerova Z, Lance S, Gould LH, Wise M, Jackson BR. 2017. Multistate outbreak of *Listeria monocytogenes* infections linked to whole apples used in commercially produced, prepackaged caramel apples: United States, 2014–2015. Epidemiol Infect 145:848–856. 10.1017/S0950268816003083. 28065170 PMC6542465

[7] Nadon C, Van Walle I, Gerner-Smidt P, Campos J, Chinen I, Concepcion-Acevedo J, Gilpin B, Smith AM, Kam KM, Perez E, Trees E, Kubota K, Takkinen J, Nielsen EM, Carleton H, FWD-NEXT Expert Panel. 2017. PulseNet International: vision for the implementation of whole genome sequencing (WGS) for global food-borne disease surveillance. Eurosurveillance 22. 10.2807/1560-7917.ES.2017.22.23.30544. PMC5479977 28662764

[8] Jagadeesan B, Baert L, Wiedmann M, Orsi RH. 2019. Comparative analysis of tools and approaches for source tracking *Listeria monocytogenes* in a food facility using whole-genome sequence data. Front Microbiol 10:947. 10.3389/fmicb.2019.00947. 31143162 PMC6521219

[9] Moura A, Tourdjman M, Leclercq A, Hamelin E, Laurent E, Fredriksen N, Van Cauteren D, Bracq-Dieye H, Thouvenot P, Vales G, Tessaud-Rita N, Maury MM, Alexandru A, Criscuolo A, Quevillon E, Donguy M-P, Enouf V, de Valk H, Brisse S, Lecuit M. 2017. Real-time whole-genome sequencing for surveillance of *Listeria monocytogenes*. Emerg Infect Dis 23:1462–1470. 10.3201/eid2309.170336. 28643628 PMC5572858

[10] Pightling AW, Pettengill JB, Luo Y, Baugher JD, Rand H, Strain E. 2018. Interpreting whole-genome sequence analyses of foodborne bacteria for regulatory applications and outbreak investigations. Front Microbiol 9:1482. 10.3389/fmicb.2018.01482. 30042741 PMC6048267

[11] Stessl B, Rückerl I, Wagner M. 2014. Multilocus sequence typing (MLST) of *Listeria monocytogenes*. Jordan K., Fox E., Wagner M. (ed). Listeria monocytogenes. Methods in Molecular Biology (Methods and Protocols). Humana Press, New York, New York. 10.1007/978-1-4939-0703-8_6 24792549

[12] Chen Y, Chen Y, Pouillot R, Dennis S, Xian Z, Luchansky JB, Porto-Fett ACS, Lindsay JA, Hammack TS, Allard M, Van Doren JM, Brown EW. 2020. Genetic diversity and profiles of genes associated with virulence and stress resistance among isolates from the 2010-2013 interagency *Listeria monocytogenes* market basket survey. PLoS One 15:e0231393. 10.1371/journal.pone.0231393. 32352974 PMC7192433

[13] Hurley D, Luque-Sastre L, Parker CT, Huynh S, Eshwar AK, Nguyen SV, Andrews N, Moura A, Fox EM, Jordan K, Lehner A, Stephan R, Fanning S. 2019. Whole-genome sequencing-based characterization of 100 *Listeria monocytogenes* isolates collected from food processing environments over a four-year period. mSphere 4:e00252-19. 10.1128/mSphere.00252-19. 31391275 PMC6686224

[14] Kim SW, Haendiges J, Keller EN, Myers R, Kim A, Lombard JE, Karns JS, Van Kessel JAS, Haley BJ. 2018. Genetic diversity and virulence profiles of *Listeria monocytogenes* recovered from bulk tank milk, milk filters, and milking equipment from dairies in the United States (2002 to 2014). PLoS One 13:e0197053. 10.1371/journal.pone.0197053. 29742151 PMC5942804

[15] Maury MM, Tsai Y-H, Charlier C, Touchon M, Chenal-Francisque V, Leclercq A, Criscuolo A, Gaultier C, Roussel S, Brisabois A, Disson O, Rocha EPC, Brisse S, Lecuit M. 2016. Uncovering *Listeria monocytogenes* hypervirulence by harnessing its biodiversity. Nat Genet 48:308–313. 10.1038/ng.3501. 26829754 PMC4768348

[16] Nielsen EM, Björkman JT, Kiil K, Grant K, Dallman T, Painset A, Amar C, Roussel S, Guillier L, Félix B, Rotariu O, Perez-Reche F, Forbes K, Strachan N. 2017. Closing gaps for performing a risk assessment on *Listeria monocytogenes* in ready-to-eat (RTE) foods: activity 3, the comparison of isolates from different compartments along the food chain, and from humans using whole genome sequencing (WGS) analysis. EFS3 14. 10.2903/sp.efsa.2017.EN-1151.

[17] Ebner R, Stephan R, Althaus D, Brisse S, Maury M, Tasara T. 2015. Phenotypic and genotypic characteristics of *Listeria monocytogenes* strains isolated during 2011–2014 from different food matrices in Switzerland. Food Control 57:321–326. 10.1016/j.foodcont.2015.04.030.

[18] Simmons C, Stasiewicz MJ, Wright E, Warchocki S, Roof S, Kause JR, Bauer N, Ibrahim S, Wiedmann M, Oliver HF. 2014. *Listeria monocytogenes* and *Listeria* spp. contamination patterns in retail delicatessen establishments in three U.S. states. J Food Prot 77:1929–1939. 10.4315/0362-028X.JFP-14-183. 25364927

[19] Vongkamjan K, Fuangpaiboon J, Jirachotrapee S, Turner MP. 2015. Occurrence and diversity of *Listeria* spp. in seafood processing plant environments. Food Control 50:265–272. 10.1016/j.foodcont.2014.09.001.

[20] Beno SM, Stasiewicz MJ, Andrus AD, Ralyea RD, Kent DJ, Martin NH, Wiedmann M, Boor KJ. 2016. Development and validation of pathogen environmental monitoring programs for small cheese processing facilities. J Food Prot 79:2095–2106. 10.4315/0362-028X.JFP-16-241. 28221969

[21] Wang Y, Pettengill JB, Pightling A, Timme R, Allard M, Strain E, Rand H. 2018. Genetic diversity of *Salmonella* and *Listeria* isolates from food facilities. J Food Prot 81:2082–2089. 10.4315/0362-028X.JFP-18-093. 30485763

[22] Stasiewicz MJ, Oliver HF, Wiedmann M, den Bakker HC. 2015. Whole-genome sequencing allows for improved identification of persistent *Listeria monocytogenes* in food-associated environments. Appl Environ Microbiol 81:6024–6037. 10.1128/AEM.01049-15. 26116683 PMC4551262

[23] Cherifi T, Carrillo C, Lambert D, Miniai I, Quessy S, Lariviere-Gauthier G, Blais B, Fravalo P. 2018. Genomic characterization of *Listeria monocytogenes* isolates reveals that their persistence in a pig slaughterhouse is linked to the presence of benzalkonium chloride resistance genes. BMC Microbiol 18. 10.1186/s12866-018-1363-9. PMC6302515 30572836

[24] Elson R, Awofisayo-Okuyelu A, Greener T, Swift C, Painset A, Amar CFL, Newton A, Aird H, Swindlehurst M, Elviss N, Foster K, Dallman TJ, Ruggles R, Grant K. 2019. Utility of whole genome sequencing to describe the persistence and evolution of *Listeria monocytogenes* strains within crabmeat processing environments linked to two outbreaks of listeriosis. J Food Prot 82:30–38. 10.4315/0362-028X.JFP-18-206. 30702931

[25] Li Z, Perez-Osorio A, Wang Y, Eckmann K, Glover WA, Allard MW, Brown EW, Chen Y. 2017. Whole genome sequencing analyses of *Listeria monocytogenes* that persisted in a milkshake machine for a year and caused illnesses in Washington State. BMC Microbiol 17. 10.1186/s12866-017-1043-1. PMC5472956 28619007

[26] Chen Y, Luo Y, Pettengill J, Timme R, Melka D, Doyle M, Jackson A, Parish M, Hammack TS, Allard MW, Brown EW, Strain EA. 2017. Singleton sequence type 382, an emerging clonal group of *Listeria monocytogenes* associated with three multistate outbreaks linked to contaminated stone fruit, caramel apples, and leafy green salad. J Clin Microbiol 55:931–941. 10.1128/JCM.02140-16. 28053218 PMC5328462

[27] Zoellner C, Ceres K, Ghezzi-Kopel K, Wiedmann M, Ivanek R. 2018. Design elements of *Listeria* environmental monitoring programs in food processing facilities: a scoping review of research and guidance materials. Compr Rev Food Sci Food Saf 17:1156–1171. 10.1111/1541-4337.12366. 33350161

[28] Orsi RH, Jagadeesan B, Baert L, Wiedmann M. 2021. Identification of closely related *Listeria monocytogenes* isolates with no apparent evidence for a common source or location: a retrospective whole genome sequencing analysis. J Food Prot 84:1104–1113. 10.4315/JFP-20-417. 33561192

[29] Moura A, Criscuolo A, Pouseele H, Maury MM, Leclercq A, Tarr C, Björkman JT, Dallman T, Reimer A, Enouf V, Larsonneur E, Carleton H, Bracq-Dieye H, Katz LS, Jones L, Touchon M, Tourdjman M, Walker M, Stroika S, Cantinelli T, Chenal-Francisque V, Kucerova Z, Rocha EPC, Nadon C, Grant K, Nielsen EM, Pot B, Gerner-Smidt P, Lecuit M, Brisse S. 2017. Whole genome-based population biology and epidemiological surveillance of *Listeria monocytogenes*. Nat Microbiol 2:1–10. 10.1038/nmicrobiol.2016.185. PMC8903085 27723724

[30] Cabal A, Pietzka A, Huhulescu S, Allerberger F, Ruppitsch W, Schmid D. 2019. Isolate-based surveillance of *Listeria monocytogenes* by whole genome sequencing in Austria. Front Microbiol 10:2282. 10.3389/fmicb.2019.02282. 31632381 PMC6779813

[31] Ruppitsch W, Pietzka A, Prior K, Bletz S, Fernandez HL, Allerberger F, Harmsen D, Mellmann A. 2015. Defining and evaluating a core genome multilocus sequence typing scheme for whole-genome sequence-based typing of *Listeria monocytogenes*. J Clin Microbiol 53:2869–2876. 10.1128/JCM.01193-15. 26135865 PMC4540939

[32] Whitworth J. 2019. Three dead and 200 ill in Spanish Listeria outbreak. Food Safety News.

[33] Centro de Coordinación de Alertas y Emergencias Sanitarias. 2019. Informe de fin de seguimiento del brote de listeriosis.

[34] Lee S, Chen Y, Gorski L, Ward TJ, Osborne J, Kathariou S. 2018. *Listeria monocytogenes* source distribution analysis indicates regional heterogeneity and ecological niche preference among serotype 4b clones. mBio 9:e00396-18. 10.1128/mBio.00396-18. 29666282 PMC5904418

[35] Koopmans MM, Brouwer MC, Bijlsma MW, Bovenkerk S, Keijzers W, van der Ende A, van de Beek D. 2013. *Listeria monocytogenes* sequence type 6 and increased rate of unfavorable outcome in meningitis: epidemiologic cohort study. Clin Infect Dis 57:247–253. 10.1093/cid/cit250. 23592828

[36] Allam M, Tau N, Smouse SL, Mtshali PS, Mnyameni F, Khumalo ZTH, Ismail A, Govender N, Thomas J, Smith AM. 2018. Whole-genome sequences of *Listeria monocytogenes* sequence type 6 isolates associated with a large foodborne outbreak in South Africa, 2017 to 2018. Genome Announc 6:e00538-18. 10.1128/genomeA.00538-18. 29930052 PMC6013608

[37] Gözel B, Monney C, Aguilar-Bultet L, Rupp S, Frey J, Oevermann A. 2019. Hyperinvasiveness of *Listeria monocytogenes* sequence type 1 is independent of lineage I-specific genes encoding internalin-like proteins. MicrobiologyOpen 8. 10.1002/mbo3.790. PMC6612545 30656829

[38] Sotgiu G, Muresu N, Dettori M, Mura E, Cossu A, Dolores Masia M, Murgia P, Cocuzza C, De Santis E, Scarano C, Spanu C, Piana A. 2018. A case of *Listeria monocytogenes* ST-219 meningo-encephalitis. IJERPH 16:8. 10.3390/ijerph16010008. 30577534 PMC6339192

[39] Chen Y, Gonzalez-Escalona N, Hammack TS, Allard MW, Strain EA, Brown EW. 2016. Core genome multilocus sequence typing for identification of globally distributed clonal groups and differentiation of outbreak strains of *Listeria monocytogenes*. Appl Environ Microbiol 82:6258–6272. 10.1128/AEM.01532-16. 27520821 PMC5068157

[40] Nightingale KK, Windham K, Martin KE, Yeung M, Wiedmann M. 2005. Select *Listeria monocytogenes* subtypes commonly found in foods carry distinct nonsense mutations in *inlA*, leading to expression of truncated and secreted internalin A, and are associated with a reduced invasion phenotype for human intestinal epithelial cells. Appl Environ Microbiol 71:8764–8772. 10.1128/AEM.71.12.8764-8772.2005. 16332872 PMC1317312

[41] Nightingale KK, Ivy RA, Ho AJ, Fortes ED, Njaa BL, Peters RM, Wiedmann M. 2008. inlA premature stop codons are common among *Listeria monocytogenes* isolates from foods and yield virulence-attenuated strains that confer protection against fully virulent strains. Appl Environ Microbiol 74:6570–6583. 10.1128/AEM.00997-08. 18791029 PMC2576717

[42] Chenal-Francisque V, Lopez J, Cantinelli T, Caro V, Tran C, Leclercq A, Lecuit M, Brisse S. 2011. Worldwide distribution of major clones of *Listeria monocytogenes*. Emerg Infect Dis 17:1110–1112. 10.3201/eid/1706.101778. 21749783 PMC3358213

[43] Van Stelten A, Simpson JM, Ward TJ, Nightingale KK. 2010. Revelation by single-nucleotide polymorphism genotyping that mutations leading to a premature stop codon in *inlA* are common among *Listeria monocytogenes* isolates from ready-to-eat foods but not human listeriosis cases. Appl Environ Microbiol 76:2783–2790. 10.1128/AEM.02651-09. 20208021 PMC2863446

[44] Van Stelten A, Roberts AR, Manuel CS, Nightingale KK. 2016. *Listeria monocytogenes* isolates carrying virulence-attenuating mutations in internalin A are commonly isolated from ready-to-eat food processing plant and retail environments. J Food Prot 79:1733–1740. 10.4315/0362-028X.JFP-16-145. 28221857

[45] Manuel CS, Van Stelten A, Wiedmann M, Nightingale KK, Orsi RH. 2015. Prevalence and distribution of *Listeria monocytogenes inlA* alleles prone to phase variation and *inlA* alleles with premature stop codon mutations among human, food, animal, and environmental isolates. Appl Environ Microbiol 81:8339–8345. 10.1128/AEM.02752-15. 26407886 PMC4644647

[46] Cotter PD, Draper LA, Lawton EM, Daly KM, Groeger DS, Casey PG, Ross RP, Hill C. 2008. Listeriolysin S, a novel peptide haemolysin associated with a subset of lineage I *Listeria monocytogenes*. PLoS Pathog 4:e1000144. 10.1371/journal.ppat.1000144. 18787690 PMC2522273

[47] Roberts A, Nightingale K, Jeffers G, Fortes E, Kongo JM, Wiedmann M. 2006. Genetic and phenotypic characterization of *Listeria monocytogenes* lineage III. Microbiology (Reading) 152:685–693. 10.1099/mic.0.28503-0. 16514149

[48] Orsi RH, den Bakker HC, Wiedmann M. 2011. *Listeria monocytogenes* lineages: genomics, evolution, ecology, and phenotypic characteristics. Int J Med Microbiol 301:79–96. 10.1016/j.ijmm.2010.05.002. 20708964

[49] Liao J, Guo X, Weller DL, Pollak S, Buckley DH, Wiedmann M, Cordero OX. 2021. Nationwide genomic atlas of soil-dwelling *Listeria* reveals effects of selection and population ecology on pangenome evolution. Nat Microbiol 6:1021–1030. 10.1038/s41564-021-00935-7. 34267358

[50] Hein I, Klinger S, Dooms M, Flekna G, Stessl B, Leclercq A, Hill C, Allerberger F, Wagner M. 2011. Stress Survival Islet 1 (SSI-1) Survey in *Listeria monocytogenes* reveals an insert common to *Listeria innocua* in sequence type 121 *L. monocytogenes* strains. Appl Environ Microbiol 77:2169–2173. 10.1128/AEM.02159-10. 21239547 PMC3067325

[51] Hingston P, Chen J, Dhillon BK, Laing C, Bertelli C, Gannon V, Tasara T, Allen K, Brinkman FSL, Truelstrup HL, Wang S. 2017. Genotypes associated with *Listeria monocytogenes* isolates displaying impaired or enhanced tolerances to cold, salt, acid, or desiccation stress. Front Microbiol 8. 10.3389/fmicb.2017.00369. PMC5340757 28337186

[52] Camargo AC, Moura A, Avillan J, Herman N, McFarland AP, Sreevatsan S, Call DR, Woodward JJ, Lecuit M, Nero LA. 2019. Whole-genome sequencing reveals *Listeria monocytogenes* diversity and allows identification of long-term persistent strains in Brazil. Environ Microbiol 21:4478–4487. 10.1111/1462-2920.14726. 31251828 PMC7644123

[53] Toledo V, den Bakker H, Hormazábal J, González-Rocha G, Bello-Toledo H, Toro M, Moreno-Switt A. 2018. Genomic diversity of *Listeria monocytogenes* isolated from clinical and non-clinical samples in Chile. Genes 9:396. 10.3390/genes9080396. 30072604 PMC6115834

[54] Nowak J, Cruz CD, Tempelaars M, Abee T, van Vliet AHM, Fletcher GC, Hedderley D, Palmer J, Flint S. 2017. Persistent *Listeria monocytogenes* strains isolated from mussel production facilities form more biofilm but are not linked to specific genetic markers. Int J Food Microbiol 256:45–53. 10.1016/j.ijfoodmicro.2017.05.024. 28599174

[55] Palaiodimou L, Fanning S, Fox EM. 2021. Genomic insights into persistence of *Listeria* species in the food processing environment. J Appl Microbiol 131:2082–2094. 10.1111/jam.15089. 33768629

[56] Lappi VR, Thimothe J, Walker J, Bell J, Gall K, Moody MW, Wiedmann M. 2004. Impact of intervention strategies on *Listeria* contamination patterns in crawfish processing plants: a longitudinal study. J Food Prot 67:1163–1169. 10.4315/0362-028X-67.6.1163. 15222544

[57] Orsi RH, Borowsky ML, Lauer P, Young SK, Nusbaum C, Galagan JE, Birren BW, Ivy RA, Sun Q, Graves LM, Swaminathan B, Wiedmann M. 2008. Short-term genome evolution of *Listeria monocytogenes* in a non-controlled environment. BMC Genomics 9:539. 10.1186/1471-2164-9-539. 19014550 PMC2642827

[58] Belias A, Sullivan G, Wiedmann M, Ivanek R. 2022. Factors that contribute to persistent *Listeria* in food processing facilities and relevant interventions: a rapid review. Food Control 133:108579. 10.1016/j.foodcont.2021.108579.

[59] EFSA Panel on Biological Hazards (EFSA BIOHAZ Panel). Whole genome sequencing and metagenomics for outbreak investigation, source attribution and risk assessment of food-borne microorganisms. EFSA J 78. 10.2903/j.efsa.2019.5898 PMC7008917 32626197

[60] Harrand AS, Jagadeesan B, Baert L, Wiedmann M, Orsi RH. 2020. Evolution of *Listeria monocytogenes* in a food processing plant involves limited single-nucleotide substitutions but considerable diversification by gain and loss of prophages. e02493–19. Appl Environ Microbiol 86. 10.1128/AEM.02493-19. PMC7054086 31900305

[61] Estrada E, Hamilton AM, Sullivan GB, Wiedmann M, Critzer FJ, Strawn LK. 2020. Prevalence, persistence and diversity of *Listeria monocytogenes* and *Listeria* species in produce packinghouses in three U.S. states. J Food Prot 83:277–286. 10.4315/0362-028X.JFP-19-411. 31961227

[62] Sullivan G, Wiedmann M. 2020. Detection and prevalence of *Listeria* in US produce packinghouses and fresh-cut facilities. J Food Prot 83:1656–1666. 10.4315/JFP-20-094. 32421820

[63] Liao J, Wiedmann M, Kovac J. 2017. Genetic stability and evolution of the *sigB* allele, used for *Listeria* sensu stricto subtyping and phylogenetic inference. Appl Environ Microbiol 83:e00306-17. 28389543 10.1128/AEM.00306-17 PMC5452827

[64] Sullivan G, Orsi RH, Estrada E, Strawn L, Wiedmann M. 2022. Data from: whole genome sequencing-based characterization of Listeria isolates from produce packinghouses and fresh-cut facilities suggests both persistence and re-introduction of fully virulent L. monocytogenes. Cornell University eCommons Repository. 10.7298/74sp-fg52. PMC9680643 36286532

[65] Bolger AM, Lohse M, Usadel B. 2014. Genome analysis Trimmomatic: a flexible trimmer for Illumina sequence data. Bioinformatics 30:2114–2120. 10.1093/bioinformatics/btu170. 24695404 PMC4103590

[66] Andrews S. 2019. FastQC. Babraham Bioinformatics. http://www.bioinformatics.babraham.ac.uk/projects/fastqc/.

[67] Bankevich A, Nurk S, Antipov D, Gurevich AA, Dvorkin M, Kulikov AS, Lesin VM, Nikolenko SI, Pham SON, Prjibelski AD, Pyshkin AV, Sirotkin AV, Vyahhi N, Tesler G, Alekseyev MAXA, Pevzner PA. 2012. SPAdes: a new genome assembly algorithm and its applications to single-cell sequencing. J Comput Biol 19:455–477. 10.1089/cmb.2012.0021. 22506599 PMC3342519

[68] Gardner SN, Slezak T, Hall BG. 2015. Sequence analysis kSNP3. 0: SNP detection and phylogenetic analysis of genomes without genome alignment or reference genome. Bioinformatics 31:2877–2878. 10.1093/bioinformatics/btv271. 25913206

[69] Davis S, Pettengill JB, Luo Y, Payne J, Shpuntoff A, Rand H, Strain E. 2015. CFSAN SNP Pipeline: an automated method for constructing SNP matrices from next-generation sequence data. PeerJ Computer Science 1:e20. 10.7717/peerj-cs.20.

[70] Stamatakis A. 2014. RAxML version 8: a tool for phylogenetic analysis and post-analysis of large phylogenies. Bioinformatics 30:1312–1313. 10.1093/bioinformatics/btu033. 24451623 PMC3998144

[71] Ragon M, Wirth T, Hollandt F, Lavenir R, Lecuit M, Monnier AL, Brisse S. 2008. A new perspective on *Listeria monocytogenes* evolution. PLoS Pathog 4:e1000146. 10.1371/journal.ppat.1000146. 18773117 PMC2518857

[72] Harter E, Wagner EM, Zaiser A, Halecker S, Wagner M, Rychli K. 2017. Stress survival islet 2, predominantly present in *Listeria monocytogenes* strains of sequence type 121, is involved in the alkaline and oxidative stress responses. Appl Environ Microbiol 83. 10.1128/AEM.00827-17. PMC5541211 28625982

[73] Ryan S, Begley M, Hill C, Gahan CGM. 2010. A five-gene stress survival islet (SSI-1) that contributes to the growth of *Listeria monocytogenes* in suboptimal conditions. J Appl Microbiol 109:984–995. 10.1111/j.1365-2672.2010.04726.x. 20408910

[74] Kumar S, Stecher G, Li M, Knyaz C, Tamura K. 2018. MEGA X: molecular evolutionary genetics analysis across computing platforms. Mol Biol Evol 35:1547–1549. 10.1093/molbev/msy096. 29722887 PMC5967553

[75] Letunic I, Bork P. 2007. Interactive Tree Of Life (iTOL): an online tool for phylogenetic tree display and annotation. Bioinformatics 23:127–128. 10.1093/bioinformatics/btl529. 17050570

